# New Appliances and Things Medical

**Published:** 1893-09-02

**Authors:** 


### NEW APPLIANCES AND THINGS MEDICAL.

[All preparations, appliances, novelties, &c., of which a notice is desired, should be sent for the Editor, to care of The Manager, 428, Strand, London, W.C.]

### NEW PREPARATIONS FROM THE SANITAS COMPANY.

We have received from the above company two forms of Kingzett's Sulphur Fumigating Candle. One, the cheaper, burns the sulphur from a central wick in a tin mould. The other has the tin mould surrounded by an outer deep rim for water. This not only produces sulphur dioside by its combustion, but produces it in company with water vapour, and so in its most efficacious form. These are two of the most useful and handy methods we have yet met with for disinfection by means of burning sulphur. Another new production is the "Sanitas" Pocket Inhaler. This is a little glass tube with an absorbent material inside. The absorbent is charged with "Sanitas Eucalyptus Oil" by means of a teat drop tube. The whole is retailed at a shilling. We have no doubt that the inhaler will be found useful in the many catarrhal complaints for which balsamic inhalations are indicated. The sulphur candles are retailed at 6d. and 1s.

